# Increased Intestinal Absorption of Vitamin U in Steamed Graviola Leaf Extract Activates Nicotine Detoxification

**DOI:** 10.3390/nu11061334

**Published:** 2019-06-14

**Authors:** Eun-Hye Choi, Seon-Bong Lee, Da-Yeon Lee, Goon-Tae Kim, Soon-Mi Shim, Tae-Sik Park

**Affiliations:** 1 Department of Food Science and Technology, Sejong University, 98 Gunja-dong, Gwangjin-gu, Seoul 05006, Korea; jweslh@naver.com (E.-H.C.); tjs4210@gmail.com (S.-B.L.); dayeons93@naver.com (D.-Y.L.); 2 Department of Life Science, Gachon University, Bokjung-dong, Sujung-gu, Sungnam, Gyeonggi-do, Seongnam 13120, Korea; chng00@naver.com

**Keywords:** graviola, excipient ingredient, vitamin U, bioavailability, nicotine

## Abstract

Graviola leaves contain much vitamin U (vit U), but their sensory quality is not good enough for them to be developed as food ingredients. Addition of excipient natural ingredients formulated alongside vit U as active ingredients could enhance not only its sensory quality but also its bioavailability. The objectives of this study were to measure the bioaccessibility and intestinal cellular uptake of bioactive components, including rutin, kaempferol-rutinoside, and vit U, from steamed extract of graviola leaves (SGV) and SGV enriched with kale extract (SGK), and to examine how much they can detoxify nicotine in HepG2 cells. The bioaccessibility of vit U from SGV and SGK was 82.40% and 68.03%, respectively. The cellular uptake of vit U in SGK by Caco-2 cells was higher than that in SGV. Cotinine content converted from nicotine in HepG2 cells for 120 min was 0.22 and 0.25 μg/mg protein in 50 μg/mL of SGV and SGK, respectively, which were 2.86 and 3.57 times higher than the no-treatment control. SGK treatment of HepG2 cells upregulated CYP2A6 three times as much as did that of SGV. Our results suggest that graviola leaf extract enriched with excipient ingredients such as kale could improve vit U absorption and provide a natural therapy for detoxifying nicotine.

## 1. Introduction

Graviola, known as *Annona muricata,* is a traditional plant throughout tropical countries including Central and South America and West Africa [1]. It is well known to have beneficial effects against cancer, diabetes, hepatic dysfunction, gastrointestinal dysfunction, and inflammation [1,2,3,4,5,6]. Graviola leaves contain various phytochemicals, including acetogenins, flavonols, and megastigmanes [7]. Recently, we found that steamed extract of graviola leaves (SGV) contain rutin, kaempferol-rutinoside, and sulfur-methylmethionine, which provide beneficial effects, such as scavenging free radicals and upregulating antioxidant genes [8]. 

As a natural sulfur-containing amino acid, sulfur-methylmethionine is called vitamin U (vit U) because it is a vitamin-like substance [9,10,11]. It originates from the *Brassica* species in the mustard family (*Brassicaceae*) and belongs to a group of physiologically active natural compounds [12,13]. Previous studies have demonstrated that vit U prevents ulcers, inflammation, and liver damage, and accelerates wound healing in skin [14,15,16,17]. Among the various natural sources of vit U, kale has the highest vit U content [18]. Kale (*Brassica oleracea var. sabellica*) is known to be an antioxidant source against peroxyl radicals as well as a protectant of bioactive phytochemicals [19]. Kale’s high content of various flavonoids provides beneficial effects, such as protection against inflammation, bacterial infection, and vascular dysfunction [20,21]. 

Smoking is the major cause of oxidative stress and generates reactive oxygen species (ROS), which cause cancer to develop in the lung, brain, and liver [22,23,24,25]. Our recent finding demonstrated that the extract of graviola leaves prevents oxidative damage of hepatocytes caused by hydrogen peroxide (H_2_O_2_) [8]. Rutin, one of the major components in graviola leaves, accelerates detoxification of nicotine to cotinine, as reported previously [26]. Although it has been known that graviola leaves contain various bioactive antioxidants, there are few studies of its health benefits in nicotine detoxification.

Since the biodegradation and metabolism of bioactive molecules in a digestive system depend on the food matrix, it is necessary to compare the physiological activities of vit U in different matrix environments during digestion in the human body [27,28,29]. An excipient food is intended to increase the bioavailability of bioactive components that are co-ingested with it [30]. We investigated whether vit U can act as a natural excipient ingredient to promote bioaccessibility and digestive absorption of SGV. The fact that matrix significantly influences the bioavailability or bioactivity of vit U has been demonstrated in the in vitro digestion model system [31,32,33].

In this study, we evaluated the ability of SGV and the extract of steamed graviola leaves supplemented with vit U from kale (SGK) as excipient ingredients to detoxify nicotine and assessed vit U stability in the digestive fraction. Our goals were: (1) to assess bioaccessibility of targeted bioactive components from SGV and SGV enriched with kale extract (SGK) in Caco-2 epithelial cells, (2) to compare the degree of cytotoxicity and ROS generation prevented by SGV or SGK, and (3) to examine the cellular conversion rate of nicotine to cotinine by SGV or SGK.

## 2. Materials and Methods

### 2.1. Preparation of Steamed Extract of Graviola Leaves (SGV) and Water Extract of Kale

Graviola leaves were obtained from the Philippines to make steamed extracts as demonstrated previously [8]. To prepare the water extract of kale, fresh kale was purchased from local markets in South Korea. The freeze-dried kale was extracted 20 times with distilled water by an agitating water bath (Biofree, Seoul, South Korea) for 6 hr at 120 rpm at room temperature. The freeze-dried powder was kept at −20 °C for further experiments.

### 2.2. Preparation of Extract of Steamed Extract of Graviola Leaves Combined with Kale (SGK)

SGK was formed with the steamed extract of graviola leaves, kale extract, and vitamin C in the ratio of 100:30:1 (W:W:W) calculated with dry weight. The combination ratio was based on a preliminary study that showed the best sensory evaluation as well as antioxidant capacity. They were measured separately and mixed together evenly. The SGK was kept in a freezer at −20 °C until further analysis. Table 1 shows the amount of rutin, kaempferol-rutinoside, and vit U in SGK as they were 615.47, 700.46, and 632.79 µg/g of dry weight, respectively.

### 2.3. In Vitro Digestion Model Coupled with Caco-2 Cell Uptake

In order to examine the amounts of bioactive components accessible to the gastrointestinal (GI) tract, the method by Yang et al. and Son et al. was used with a slight modification [34,35]. The GI tract was composed of three-stages of an in vitro human digestion model system, which were salivary, stomach, and upper intestine steps. All digestive enzymes were prepared and put into ice with an approximate temperature of 4 °C. 

Caco-2 cells (Korean Cell Line Bank, Seoul, Republic of Korea) were seeded in 12-well plates (Corning, NY, USA) with 1 × 10^5^ cell per well and the epithelial cells were grown in Dulbecco’s Modified Eagle’s Medium (DMEM) supplemented with 10% fetal bovine serum (FBS) (Thermo Fisher Scientific, Waltham, MA, USA), 1% penicillin/streptomycin solution, 1% nonessential amino acids, and 0.1% gentamycin (Sigma-Aldrich, St. Louis, MO, USA) until 100% of confluence. Before treatment, the phosphate buffer solution (PBS; pH 7.0, Corning) was used for washing the cells to make the cells starve for FBS for 30 min in a 5% CO_2_ generating incubation. Then a digested aqueous fraction (0.5 mL) was dispensed into each well.

### 2.4. Analysis of Rutin, Kaempferol-Rutinoside, and vit U by UPLC-ESI-MS

As demonstrated previously [8], rutin, kaempferol-rutinoside, and vit U were identified and quantified by using ultraperformance liquid chromatography-electrospray ionization-mass spectroscopy (UPLC-ESI-MS) equipped with an Accela photodiode array (PDA), Accela auto sampler, Accela 600 pump, and an LCQ fleet (Thermo Scientific, Vantaa, Finland). A gradient condition for analysis of rutin and kaempferol-rutinoside was as follows: a linear change from A:B (1% formic acid:acetonitrile, 95:5, v/v) to A:B (5:95, v/v) for 60 min. The injection volume was 20 μL with a 1.0 mL/min flow rate. PDA wavelength was set at 340 nm. A gradient elution of vit U was performed as follows: 0 to 3.3 min, 50–61% A; 3.3 to 4 min, 61–55% A; 4 to 5 min, 55–80% A; 5 to 7 min, 80–50% A; 7 to 10 min, 50–50% A. The flow rate was 0.2 mL/min with an injection volume of 2.0 μL.

### 2.5. Evaluation for Protective Effects against Cytotoxicity of HepG2 Celsl Induced by Nicotine

An MTT (3-(4,5-dimethylthiazol-2-yl)-2,5-diphenyltetrazolium bromide) assay was conducted to measure the protective effects of cytotoxicity of HepG2 cells treated with SGV and SGK prior to induction by 1 mM nicotine. The average values of cell viability were calculated as follows at 570 nm of optical density (OD):

$$\mathrm{cell}\;\mathrm{viability}\;\left(\%\right)=\frac{\mathrm{OD}\;\left(\mathrm{Average}\;\mathrm{of}\;\mathrm{sample}\;-\;\mathrm{Average}\;\mathrm{of}\;\mathrm{blank}\right)\;}{\mathrm{OD}\;\left(\mathrm{Average}\;\mathrm{of}\;\mathrm{control}-\;\mathrm{Average}\;\mathrm{of}\;\mathrm{blank}\right)}\times 100$$

### 2.6. Evaluation of Inhibiting Reactive Oxygen Species (ROS) of HepG2 Cells Induced by Nicotine

The inhibition of oxidative damage on cell growth was measured by generation of fluorescent 2′,7′-dichlorofluorscein (DCF) from the reaction of 2′,7′-dichlorofluorescin diacetate (DCFH-DA) and ROS radicals, as described previously with modifications [36]. The fluorescence of cells was measured by using the microplate reader (Varioskan Flash, Thermo Scientific, San Jose, CA, USA) at 488 nm for excitation and at 525 nm for emission. The means of ROS generation (%) were calculated as follows:

$$\mathrm{ROS}\;\mathrm{generation}\left(\%\right)=\frac{\mathrm{OD}\left(\mathrm{Average}\;\mathrm{of}\;\mathrm{sample}\right)}{\mathrm{OD}\left(\mathrm{Average}\;\mathrm{of}\;\mathrm{control}\right)}\times 100$$

### 2.7. Assessment of Cotinine Contents in HepG2 Cells Converted from Nicotine by SGV and SGK

The contents of cotinine converted from nicotine were quantified in HepG2 cells, which help activate enzymes and detoxify the liver. The direct barbiturate assay (DBA) was conducted to measure the amount of cotinine contents in HepG2 cells with reference to Kim et al. [37]. To measure cell protein in cells, the Bradford assay was used. Cells were homogenized by using a cell lysis buffer and then centrifuged at 13,000 rpm for 5 min to collect the supernatant. A total of 2 μL of the supernatant, 100 μL of Bradford reagent, and 8 μL of distilled water were mixed. Its absorbance was detected at 595 nm by using a multi microplate reader.

### 2.8. RNA Isolation and Real-Time RT-PCR

HepG2 cells were obtained from the American Type Culture Collection (ATCC, Manassas, VA, USA). Cells were cultured in high glucose Dulbecco’s Modified Eagle’s Medium (DMEM) supplemented with 10% fetal bovine serum (FBS), 100 U/mL of penicillin, and 100 μg/mL of streptomycin under a 5% CO_2_ humidified atmosphere at 37 °C. For treatments of SGV and SGK, HepG2 cells were seeded at 8 × 10^5^ cells/well in a 6-well culture plate and incubated for 24 h. At first, the cells were pretreated with the two extracts at various concentrations. When the cells were pretreated for 4 h, they were then treated with 1 mM nicotine for 24 h.

Total RNAs were isolated from HepG2 cells using the RNA Extraction Kit (iNtrRON Biotechnology, Sungnam, Korea) according to the manufacturer’s instructions. Real-time PCR analysis was performed in the Step One Plus Real-Time PCR System (Applied Biosystems, Foster City, CA, USA) using SYBR Green Master Mix (TaKaRa, Mountain View, CA, USA). 

### 2.9. Statistical Analysis

Results are presented as representative data from triplicate sets of experiments. Data are expressed as the mean ± standard error of the mean (SEM). Statistical analysis for comparison among groups was performed using analysis of variance (ANOVA) followed by Tukey’s post hoc test using Graphpad Prism 3.0 software (Graphpad, La Jolla, CA, USA). A difference between means was considered statistically significant at *p* < 0.05.

Detailed methods are provided in the **Supplementary Materials**.

## 3. Results and Discussion

### 3.1. Bioaccessibility of Rutin, Kaempferol-Rutinoside, and Vit U in SGV and SGK

**Figure 1:**
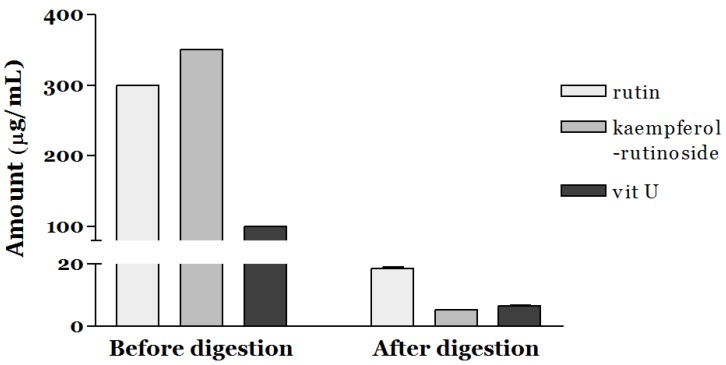
Bioaccessibility of bioactive components including rutin, kaempferol-rutinoside, and vitamin U (vit U) after simulated gastrointestinal digestion.

**Figure 2:**
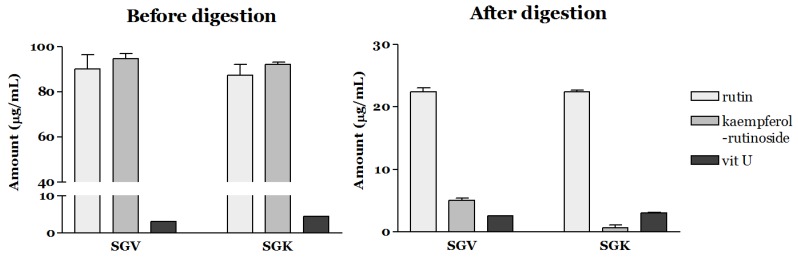
Bioaccessibility of rutin, kaempferol-rutinoside, and vit U from SGV (steamed extract of graviola leaves) and SGK (SGV enriched with kale extract) after simulated gastrointestinal digestion.

Since the bioaccessibility of a commercial grade of standard compounds such as rutin, kaempferol-rutinoside, and vit U is poor, we assessed the bioaccessible amount (μg/mL) of the targeted bioactive components in SGV and SGK using in vitro simulated gastrointestinal digestion (Figure 1). At the ratio of 6:7:2 (W:W:W), the amount of rutin, kaempferol-rutinoside, and vit U were 300, 350, and 100 μg/mL, respectively. Only 6.18% of the initial amount of rutin (18.54 μg/mL) was stable during digestion, and a similar pattern was observed for vit U (6.55% of bioaccessibility). Furthermore, the bioaccessibility of kaempferol-rutinoside was significantly lower (1.49%) than that of the others (*p* < 0.05). Figure 2 shows the bioaccessibility of rutin, kaempferol-rutinoside, and vit U from SGV and SGK. SGV contained 3.17, 90.20, and 94.59 μg/mL of vit U, kaempferol-rutinoside, and rutin, respectively, and vit U was the most bioaccessible compound (2.61 μg/mL) followed by rutin (22.37 μg/mL), and kaempferol-rutinoside (5.08 μg/mL) after digestion. Thus, the aqueous fraction from oral intake (bioaccessibility, %) was 82.40, 24.91, and 5.37% for vit U, rutin, and kaempferol-rutinoside, respectively. For SGK, the quantified bioaccessible fraction was 3.06 μg/mL, 22.39 μg/mL, and 0.72 μg/mL, and for vit U, rutin, and kaempferol-rutinoside, the bioaccessibility was 68.03, 25.72, and 0.78%, respectively. In general, both extracts showed higher bioaccessibility than did the standards of the targeted bioactive component itself. This result suggests that the digestive stability of bioactive components in a food matrix was considerably higher than that of standard material alone. The bioaccessibility of rutin from SGV and SGK was 4.03 and 4.16 times higher than that of the standard alone, respectively. Even though kaempferol-rutinoside from SGK had 0.52 times lower bioaccessibility than did standard kaempferol-rutinoside, a bioaccessible ratio 3.60 times higher for kaempferol-rutinoside from SGV was observed when compared to that of the standard alone. To the extent of bioaccessibility of vit U, both the digested SGV and the SGK extracts had 12.58 and 10.39 times higher amounts than did only the vit U standard.

The bioaccessibility of rutin from blended juice containing various fruits to increase vitamin C was found to be 22.16%, which was 1.12 and 1.16 times lower than that of SGV and SGK, respectively [38]. A previous study demonstrated that the bioaccessibility of vit U digested from Kimchi cabbage was higher than that of vit U alone [33]. In a similar finding, the study reported that 8.83%, 14.71%, and 10.88% of bioaccessibility were found for salivary, gastric, and small intestinal parts. It is a comparable finding that the bioaccessibility of vit U from SGV and SGK was 7.47 and 6.33 times higher than that of Kimchi cabbage after the upper small intestinal steps. Miranda et al. reported that kaempferol-rutinoside in two different cultivars of potatoes was not detectable after gastrointestinal digestion [39]. The results suggest that the digestibility of SGV and SGK was outstanding compared with that of other food materials.

### 3.2. Intestinal Uptake of Rutin, Kaempferol-Rutinoside, and vit U from SGV and SGK

**Figure 3:**
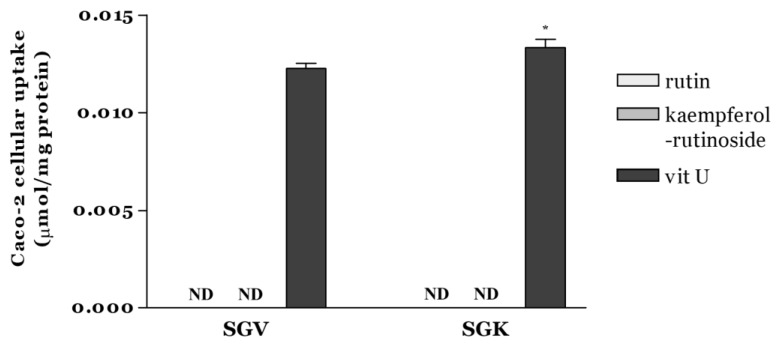
Caco-2 cellular uptake of rutin, kaempferol-rutinoside, and vit U from SGV and SGK after 2 h incubation expressed as µmol/mg protein. * indicates a significant difference between samples (*p* < 0.05). ND (Not detectable): amount below the limit of detection.

Intestinal cellular uptake of rutin, kaempferol-rutinoside, and vit U from SGV and SGK was estimated by means of Caco-2 cells after digestion (Figure 3). Among the targeted bioactive components, quantified vit U in cells treated with SGV or SGK was 0.0123 ± 0.0003 and 0.0133 ± 0.0004 µmol/mg protein, respectively. It was significantly different between SGV and SGK (*p* < 0.05). However, rutin and kaempferol-rutinoside were not detectable in either extract.

Similar to our findings, it was observed that rutin, known as quercetin-3-*O*-rutinoside, was not absorbed well in the small intestine of rats [40]. The study also reported that rutin was not digested easily in the small intestine but was digested mostly in the large intestine by large intestinal microbiota. Serra et al. found that the metabolism of rutin and kaempferol-rutinoside after gastrointestinal digestion and their metabolites were detected as some forms of acids in a colonic fermentation model [41]. Generally, it is well known that polyphenol uptake is very poor in the small intestine and is hydrolyzed in the colon with microflora for absorption. Especially, polyphenol glycosides were rarely absorbed in the small intestine and could be absorbed only as the forms of aglycones. Therefore, rutin and kaempferol-rutinoside, in the forms of glycosides, could not be accumulated in Caco-2 cells. As a derivative of methionine, vit U was absorbed into Caco-2 cells according to time, concentration, and temperature, and the amount of vit U uptake increased proportionally depending on these factors [42]. Findings from our study suggest that SGK rather than SGV could provide better absorption of specific bioactive components, leading to more benefits for health.

### 3.3. Measurement of Protective Effects on HepG2 Cell Cytotoxicity Induced by Nicotine

**Figure 4:**
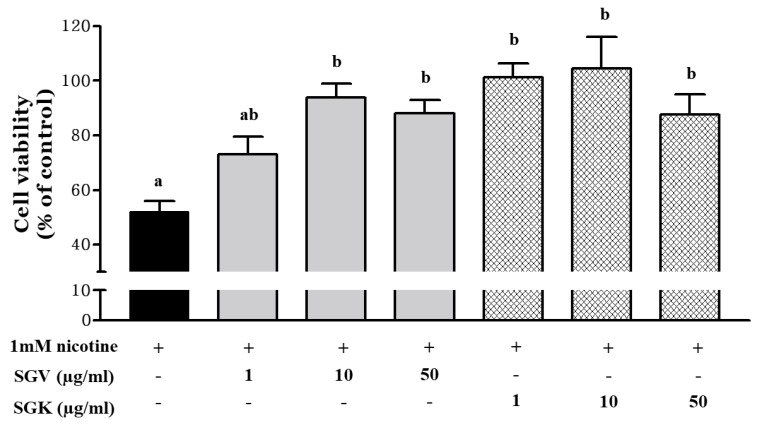
The protective effect of SGV and SGK on cell viability in HepG2 cells induced by 1 mM nicotine. Different letters show a significant difference at *p* < 0.05 between treatments. + means with treatment and - means without treatment.

We examined how SGV and SGK protect HepG2 cells against the cytotoxicity induced by nicotine (Figure 4). Cell viability decreased to 52.1% by treatment with 1 mM nicotine and reached nearly inhibitory concentration for 50% (IC_50_) of cells. Prior to IC_50_ of nicotine treatment, HepG2 cells were treated with either SGV or SGK at various concentrations. Cell viability (% of control) was 72.9%, 93.8%, and 88.0% for 1, 10, and 50 μg/mL of SGV, respectively. In addition, pretreatment with SGK, which contains kale, increased cell viability to 101.2%, 104.4%, and 87.6% at 1, 10, and 50 μg/mL concentration, respectively. These results suggest that SGK prevents the cytotoxicity caused by nicotine in HepG2 cells better than SGV does.

Our previous study reported that treatment with an ethanol extract of *Smilax china* root (EESC) in HepG2 cells prior to nicotine exposure effectively increased the cell viability up to 76% [37]. The effect of EESC containing 2.1 μmol/L of resveratrol and 0.5 μmol/L of oxyresveratrol in protecting the hepatoma cells from damage by nicotine was 1.23 and 1.37 times less than when they were treated with 10 μg/mL of SGV and SGK, respectively. The cell viability of HepG2 cells treated with ethanol extract of raw Ethiopian kale (*Brassica carinata*) not only decreased IC_50,_ but no cytotoxicity was found until their concentration reached 333.30 μg/mL [43]. Even though large amounts of kale extracts were used to treat liver cells, they did not affect cell survival. The finding that co-administration of vit U with acetaminophen (APAP), a hepatotoxicity inducer, prevented liver damage in mice was also reported [44]. We evaluated whether the other form of vit U, vit U-chloride, protects against valproic acid (VPA)-induced liver damage in rats [17]. Therefore, the inhibitory effect of nicotine on the cytotoxicity in HepG2 cells was increased by kale extracts supplemented with vit U in SGV and SGK.

### 3.4. Inhibitory Effect of SGV and SGK on Oxidative Stress Induced by Nicotine in HepG2 Cell

**Figure 5:**
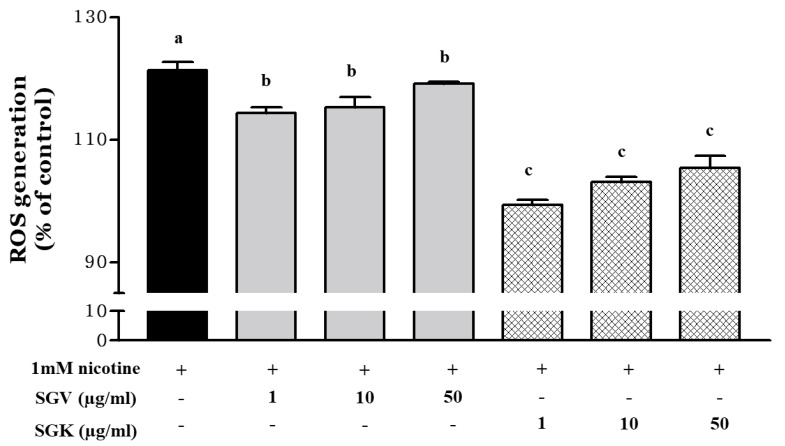
Inhibition of reactive oxygen species (ROS) generation in HepG2 cells pretreated with various concentrations of SGV and SGK prior to induction of cytotoxicity by 1 mM nicotine. Different letters represent a significant difference at *p* < 0.05. + means with treatment and - means without treatment.

Since nicotine induces generation of reactive oxygen radical species (ROS), we examined whether SGV or SGK prevents the cytotoxicity caused by nicotine by reducing ROS production (Figure 5). After HepG2 cells were treated with 1 mM nicotine, ROS production was increased by 121.4% when compared with the no-treatment control. When SGV was pretreated to the cells before nicotine treatment, ROS production was decreased by 114.3%, 115.3%, and 119.2% at 1, 10, and 50 μg SGV/mL, respectively. Although only two lower dosages of SGV resulted in significant reductions in ROS formation, all concentrations of SGK (1, 10, and 50 μg/mL) appeared to decrease the generation of ROS more than did those of SGV; the ROS was reduced to 99.4%, 103.1%, and 105.5% of the no-treatment control, respectively. Treatment with 1 μg/mL of SGK could restore ROS production to the levels of the no-treatment control. 

Previously, we found that SGV could inhibit the oxidative stress induced by H_2_O_2_ [8]. Nicotine, a constituent of tobacco leaves circulated by the blood vessels, is metabolized in the liver and produces various metabolites including nicotine-N’-oxide that could induce hepatotoxicity [45,46,47]. Some plants are abundant in polyphenols that could act as bioactive components to prevent carcinomas and oxidative stress [48,49]. *Mentha spicata* supplementation protected the liver of Wistar rats against nicotine-induced oxidative damage [50]. Therefore, the ROS-scavenging efficacy of SGV was elevated by kale supplementation and contributed to prevention of cytotoxicity by nicotine.

### 3.5. Effects of SGV and SGK on Nicotine Conversion to Cotinine in Hepatoma (HepG2) Cells

**Figure 6:**
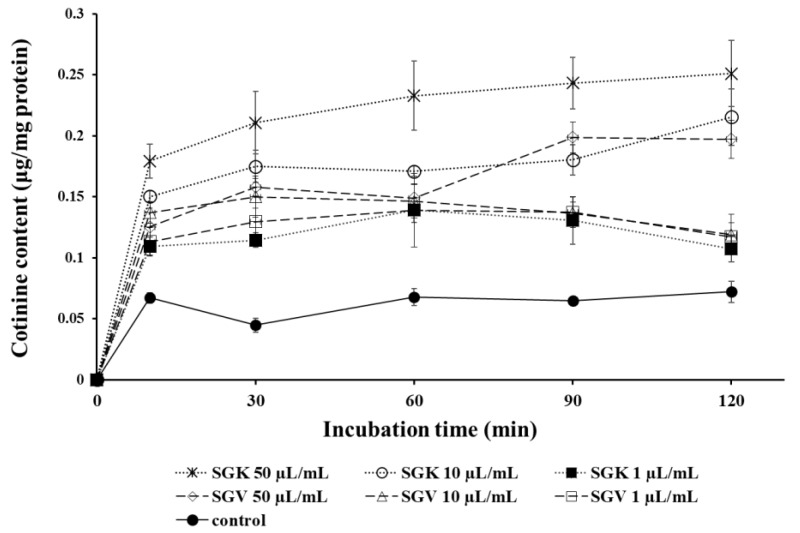
The changes of cotinine content converted from nicotine in HepG2 cells after being treated with various concentrations of SGV and SGK during 120 min, which is the half-life of nicotine.

Nicotine is metabolized in the liver and converted to cotinine. We examined whether SGV or SGK modulates the conversion rate of nicotine to cotinine. After HepG2 cells were treated with nicotine in the presence of SGV or SGK at various concentrations, cotinine levels were measured by LC/MS/MS at various times. For all treatments with SGV or SGK, we found a significant increase in cotinine levels when compared with those of the no-treatment control (Figure 6). Especially at 50 μg/mL of SGK treatment, there was a drastic increase in cotinine levels after 10 min of incubation time compared to other treatments. The conversion rate was increased to 0.18 μg/mg protein at 10 min and steadily rose to 0.25 μg/mg protein for 120 min. The second highest production of cotinine was with 10 μg/mL of SGK; it reached 0.15 μg/mg at 10 min and was sustained at 0.22 μg/mg protein at 120 min. In general, treatment with SGV and SGK accelerated detoxification of nicotine by converting nicotine to cotinine. 

Nicotine is metabolized to cotinine by CYP2A6 in the liver [51], and some plants were involved in reducing oxidation and degradation of nicotine in the liver [52]. We previously reported that HepG2 cells treated with *Smilax china* root extract (EESC) increased the conversion rate of nicotine to cotinine by resveratrol and its analog, oxyresveratrol [37]. In this study, we found a more potent efficacy of nicotine turnover, which is 2.86 and 3.57 times higher with SGV and SGK than with EESC. The hepatic enzymes involved in nicotine metabolism were elucidated; one of them was CYP2A6 [53]. The current study demonstrated that some bioactive components, such as rutin, kaempferol-rutinoside and vit U in SGV and SGK, could be involved in nicotine metabolism. Since the biotransformation of nicotine to cotinine was catalyzed by CYP2A6 in human liver microsomes [54], we questioned whether CYP2A6 is regulated by SGV or SGK.

### 3.6. Effect of SGV and SGK on Expression of CYP2A6 in HepG2 Cells

**Figure 7:**
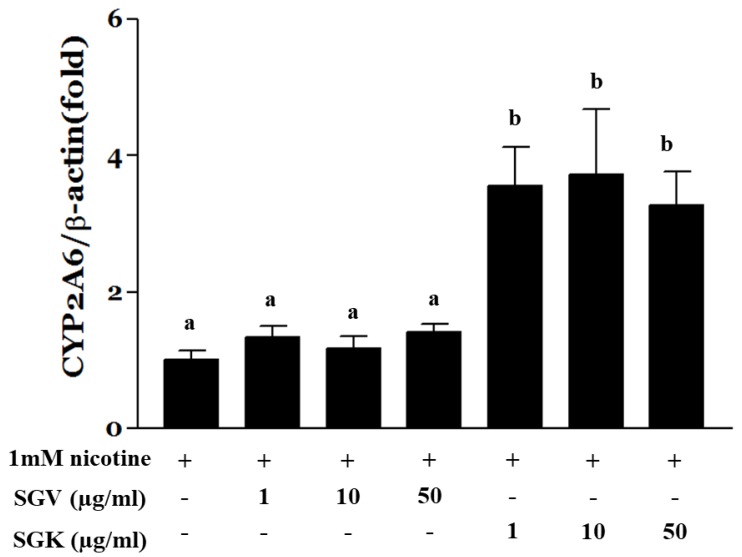
HepG2 cells were treated with 1 mM nicotine for 24 h after pre-treatment with SGV or SGK at various concentrations for 4 h. Total RNA was isolated and real-time PCR was done to measure the expression of CYP2A6. Expression was normalized to β-actin. The data were expressed by mean ± SEM. Different letters represent a significant difference at *p* < 0.05. + means with treatment and - means without treatment.

Since we found that cotinine levels were elevated by SGV or SGK, we questioned whether SGV alone or SGK modulates the expression of CYP2A6. We pre-treated the HepG2 cells with SGV or SGK at various concentrations for 4 h and treated the cells with nicotine for an additional 24 h. Then, CYP2A6 mRNA was quantified by real-time PCR to find out whether the increased cotinine by SGV and SGK is associated with alterations of CYP2A6 expression. Expression of CYP2A6 was not altered when the cells were treated with SGV only in the presence of nicotine. In contrast, CYP2A6 was upregulated significantly by 3.5-, 3.7-, and 3.2-fold when the cells were treated with 1, 10, and 50 μg/mL of SGK, respectively (Figure 7). This result suggests that SGK upregulates CYP2A6 and contributes to greater nicotine metabolism when compared with the effect of SGV.

CYP2A6 is an enzyme that metabolizes carcinogens and coumarin-type alkaloids and converts nicotine to cotinine or norcotinine in the liver [55,56,57,58]. In hepatocytes, the amounts of cotinine converted from nicotine were increased for both SGV and SGK, whereas the transcriptional expression of CYP2A6 was elevated by SGK but not by SGV. These results suggest that the components of SGK contribute to the conversion of nicotine to cotinine via transcriptional upregulation of CYP2A6. Nicotine has been reported to be a major component of tobacco addiction that binds to neuronal nicotinic acetylcholine receptors (nAChRs) and induces the release of dopamine in the nucleus accumbens [59,60]. These effects of SGV and SGK are expected to activate nicotine metabolism, which reduces nicotine while decreasing ROS production to inhibit addiction to tobacco and tumorigenesis.

The current study found that bioaccessibility and intestinal absorption of rutin, kaempferol-rutinoside, and vit U enhanced from SGV and SGK, from the food matrix, rather than single components without matrix. Especially, SGK, which is SGV supplemented with vit U, advanced the preventive effects and restoration against nicotine toxicity in HepG2 cells in vitro. Moreover, cotinine conversion rates and the expression of a metabolizing enzyme, CYP2A6, were elevated in comparison to SGV. In conclusion, vit U enriched SGK can be developed as a functional beverage to detoxify nicotine exposure with high bioavailability, as proven in a biomimicry digestion system.

## 4. Conclusions

The current study found that addition of natural ingredients formulated alongside vit U such as kale enhanced bioaccessibility and intestinal cellular uptake of bioactive components, including rutin, kaempferol-rutinoside, and vit U, from steamed extract of graviola leaves (SGV) and SGV enriched with kale extract (SGK) as well as detoxifying effect of nicotine in HepG2 cells. The results suggest that graviola leaf extract enriched with excipient natural could increase vit U absorption, providing a natural therapy for detoxifying nicotine.

## Figures and Tables

**Table 1:** Contents of bioactive components in steamed extract of graviola leaves (SGV) and SGV enriched with kale extract (SGK).

	Contents (µg/g of Dry Weight)		
	Rutin	Kaempferol-Rutinoside	Vitamin U
SGV	615.47	700.46	228.02
SGK	615.47	700.46	632.79

## Supplementary Materials

The following are available online at https://www.mdpi.com/2072-6643/11/6/1334/s1.
