# Supplementary material for: Increased Intestinal Absorption of Vitamin U in Steamed Graviola Leaf Extract Activates Nicotine Detoxification
Source: Nutrients. 2019 Jun 14;11(6):1334. doi: 10.3390/nu11061334 (PMC6627529; doi:10.3390/nu11061334)
Supplement: Supplementary file 1 [file nutrients-11-01334-s001.pdf]

nutrients

Supplementary Information

Materials and Methods

Standards and chemicals

Standards of rutin, kampferol-rutinoside, vit U having HPLC grade were obtained from Sigma Aldrich (St. Louis, MO, USA). High-performance liquid chromatography (HPLC) grade of acetonitrile and water were obtained from J.T. Baker (Phillipsburg, NJ, USA). For *in vitro* digestion enzymes, α-amylase from human saliva, and pepsin, lipase, pancreatin, and bile extract from porcine were purchased from Sigma Aldrich (St. Louis, MO, USA). Protease from bovine pancreas and Bradford reagent were obtained from Sigma Aldrich, and lysis buffer was purchased from Life technologies™ (Frederick, MD, USA).

Preparation of water extract of kale

To prepare the water extract of kale, fresh kale was purchased from local market in South Korea. After washing, 400 g of kale was freeze-dried for 5 days and ground by using Mixer (Hanil electric Co., LTD, Seoul, South Korea). After filtering using 8.0 μm filter paper (Whatman Ltd., Piscataway, NJ, USA), the extract was condensed by a rotary vacuum evaporator at 50 °C and freeze-dried for 5 days and stored at -20 °C until further analysis.

In vitro digestion model system coupled with Caco-2 cell uptake

Aliquot amount of commercial grade of standard material or 1 g of extracts was suspended in 1.5 mL of 20 mM phosphate buffer (PB). For the salivary digestion, each sample was mixed with 1 mL of α-amylase (0.016 mg/mL of 20 mM PB) and then the pH of the samples was adjusted to 6.9. The samples were incubated in a shaking water bath at 37 °C at 100 rpm for 3 min. Immediately, 2 mL of pepsin (3 mg/mL in 100 mM $NaHCO_3$) was added to build up the gastric phase and then the pH of the solution was adjusted to pH 2 with 0.1 M HCl. The samples were incubated for one hour in a shaking water bath at 37 °C at 100 rpm. For the upper small intestine phase, the pH was adjusted to 5.3 using 100 mM sodium bicarbonate solution and then 3 mL of pancreatic enzyme juice containing bile acid (2.4 mg/mL of 20 mM PB), lipase (0.2 mg/mL of 20 mM PB), and pancreatic solution (0.4 mg/mL of 20 mM PB) was added. The pH increased to pH 7 by using 0.1 M NaOH and then incubated for 2 hours at 37 °C at 100 rpm. After digestion, the samples were centrifuged at 3000 rpm for 30 min and separated the supernatants. They were stored at – 4 °C and filtered by using 0.2 μm membrane filter until liquid chromatography-mass spectroscopy (LC-MS) analysis.

Caco-2 Cells were incubated at 37 °C in 5% CO_2. Caco-2 cells were utilized after 7 to 10 days post-seeding. Before adding samples, cells were incubated with a phosphate buffer saline solution (PBS; pH 7.4) for 30 min. The samples were treated to each well and then incubated for 2 hr at 37 °C in 5% CO_2. After incubation, the test mediums were removed, and then the cells were collected by using 0.25% trypsin-EDTA and protease of 10 mg/mL in PBS. Centrifugation was carried out at 3,218 g for 5 minutes. Cell pellets were sonicated at 37 °C for 30 min and filtered through 0.2 μm membrane filter prior to LC/MS analysis.

Determination of rutin, kaempferol-rutinoside, and vit U by UPLC-ESI-MS

Rutin and kaempferol-rutinoside were separated by using a Zorbax Eclipse XDB-C_{18} column (250 × 4.6 mm, 5 μm, Agilent technologies). The mobile phase contains 1% formic acid in water (A) and, acetonitrile (B) of HPLC grade (Sigma-Aldrich). Chromatographic separation of vit U was performed on a Hypersil Gold perfluorinated phenyl (PFP) column (150 mm × 2.1 mm, 3 μm, Thermo Scientific) with a mobile phase of solvents A and B (0.1% formic acid in water and 0.1% formic acid in acetonitrile). After separation, positive mode in the range of m/z 50-300 was set for the MS analysis.

Other working MS parameters were the capillary voltage of 7000 V, capillary temperature of 200 °C, sheath gas of 29 arb, and auxiliary of 2 arb.

Evaluation for protective effect toward cytotoxicity of HepG2 cell induced by nicotine

Using the MTT for the assay, living cells including enzymes in mitochondria were dyed and estimated colorimetrically. HepG2 cells were purchased from Korean Cell Line Bank (KCLB, Seoul, Republic of Korea). After 2 to 3 subculture iterations, cells were added in a 96-well plate on a cell density of $1x10^4$ cells per well in 200 μL media and incubated at 37 °C for 24 h in a 5% CO_2. After media removal, cells were treated with various concentrations of SGV and SGK then incubated at 37 °C for 4 h. Besides, cells with no treatment were defined as the control and used to calculate cell viability. Cells were treated with 200 μM nicotine and incubated for 24 h at 37 °C. Cell viability was measured by MTT assay. Absorbance at 570 nm wavelength was measured using a microplate reader (Varioskan Flash, Thermo Scientific, San Jose, CA).

Assessment of cotinine contents in HepG2 cells converted from nicotine by SGV and SGK

HepG2 cells added in 24-well plates and grown until it reached a 100% confluence condition. The media were changed to different media containing 1 mM nicotine and incubated at 37 °C in 5% CO_2 for 24 hours. A various concentration of SGV (1, 10, 50 μL/mL) and SGK (1, 10, 50 μL/mL) were treated to HepG2 cells and estimated the content of cotinine altered from nicotine during 120 minutes which means nicotine decreased by half. The time periods for estimation were carried out at interval of 30 minutes. To collect the cells at the certain time, 0.25% trypsin-EDTA was dispensed to them and 100 μL of PBS was added. Centrifugations were worked at 230 g for 5 minutes, followed by sonicating for 30 minutes to break up the cells. 100 μL of 4 M Sodium acetate buffer (pH 4.7), 40 μL of 1.5 M Potassium cyanide, 40 μL of 0.4 M Chloramine T, and 200 μL of 78 mM barbituric acid, in acetone/water (50/50, w/w) were mixed with the supernatant for 10 seconds and laid down for 15 minutes at room temperature to react within the mixture. After inserting 40 μL of 1 M Sodium metabisulfite, the reaction had stopped and it was evaluated colorimetrically by using a microplate reader (Thermo scientific) at the absorbance of 490 nm. The cotinine contents in HepG2 cells were quantified by substituting cotinine standard curve and their protein quantities were amended through Bradford assay.

Protein assay

To measure cell protein in cells, bradford assay was used. Cells were homogenized by using a cell lysis buffer and then centrifuged at 13000 rpm for 5 minutes to collect the supernatant. 2 μL of the supernatant, 100 μL of bradford reagent, and 8 μL of distilled water were mixed. Its absorbance was detected at 595 nm by using a multi microplate reader.

RNA isolation and real-time RT-PCR

First-strand cDNA using isolated mRNA as template was synthesized in PCR Thermal Cycler (TaKaRa Bio, Japan) using iScript cDNA synthesis Kit (BioRad, Hercules, CA). The PCR conditions were 25 °C for 5 minutes, 42 °C for 30 minutes, 8 °C for 5 minutes, and then kept at 4 °C for maintenance. The real time-PCR conditions were 95 °C for 5 minutes, followed by 40 cycles of 95 °C for 15 seconds, 60 °C for 15 seconds, 72 °C for 45 seconds and finally extension at 95 °C for 15 seconds and 60 °C for 1 minute. Primer sequences used for quantification were: forward 5'-ATCGATCCCACCTTCTTCCT-3' and reverse 5'-CTAGCATCATGCGCAACAGT-3' for Cytochrome P450 2A6 (CYP2A6); forward 5'-GGCATCCTCACCCTGAAGTA-3' and reverse 5'-GGGGTGTTGAAGGTCTCAAA-3' for β-actin. Expression of the genes was normalized by the relative ratio to β-actin.
